# Implications of Regurgitative Feeding on Plastic Loads in Northern Fulmars (*Fulmarus glacialis*): A Study from Svalbard

**DOI:** 10.1021/acs.est.2c05617

**Published:** 2023-02-22

**Authors:** Felix Tulatz, Geir Wing Gabrielsen, Sophie Bourgeon, Dorte Herzke, Rupert Krapp, Magdalene Langset, Svenja Neumann, Anna Lippold, France Collard

**Affiliations:** † Department of Arctic and Marine Biology, UiT—The Arctic University of Norway, N-9037 Tromsø, Norway; ‡ Fram Centre, Norwegian Polar Institute, N-9296 Tromsø, Norway; § Fram Centre for Climate and the Environment, Fram Centre, Norwegian Institute for Air Research, N-9296 Tromsø, Norway; ∥ Norwegian Institute for Nature Research, Høgskoleringen, Trondheim 97034, Norway

**Keywords:** marine pollution, polymers, Arctic, fledglings, FTIR, parental transfer, chick-rearing, Procellariiformes, microplastic

## Abstract

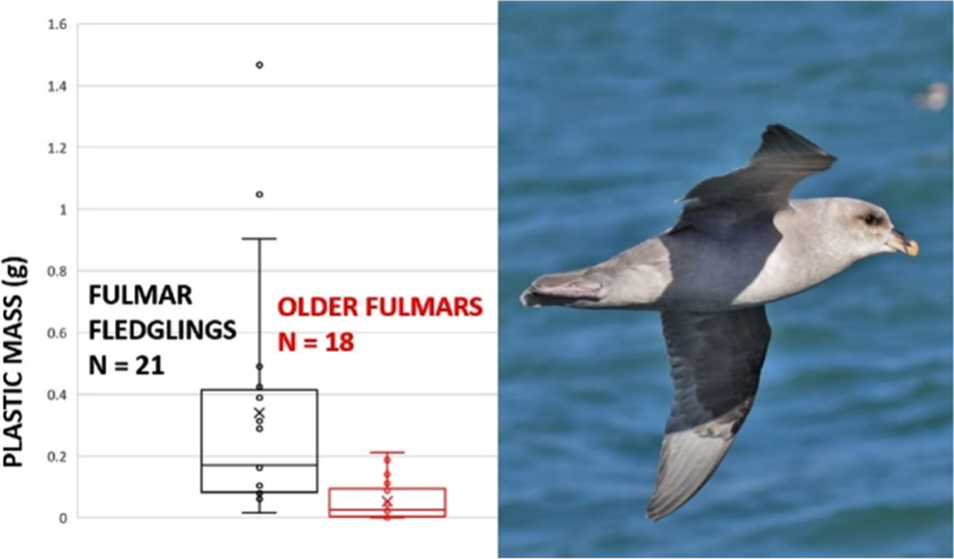

Procellariiform seabirds like northern fulmars (*Fulmarus glacialis*) are prone to ingest and accumulate floating plastic pieces. In the North Sea region, there is a long tradition to use beached fulmars as biomonitors for marine plastic pollution. Monitoring data revealed consistently lower plastic burdens in adult fulmars compared to younger age classes. Those findings were hypothesized to partly result from parental transfer of plastic to chicks. However, no prior study has examined this mechanism in fulmars by comparing plastic burdens in fledglings and older fulmars shortly after the chick-rearing period. Therefore, we investigated plastic ingestion in 39 fulmars from Kongsfjorden (Svalbard), including 21 fledglings and 18 older fulmars (adults/older immatures). We found that fledglings (50–60 days old) had significantly more plastic than older fulmars. While plastic was found in all fledglings, two older fulmars contained no and several older individuals barely any plastic. These findings supported that fulmar chicks from Svalbard get fed high quantities of plastic by their parents. Adverse effects of plastic on fulmars were indicated by one fragment that perforated the stomach and possibly one thread perforating the intestine. Negative correlations between plastic mass and body fat in fledglings and older fulmars were not significant.

## Abstract 2

No previous study has simultaneously investigated plastic ingestion by fledglings and older individuals of northern fulmars after the chick-rearing period. This study reports high burdens of plastic in fledglings (50−60 days old) from Svalbard compared to older fulmars, indicating that parental transfer of plastic is an important mechanism in this species in the Arctic.

## Introduction

Despite polar ecosystems being commonly regarded as remote and pristine, plastic pollution was documented in all marine compartments of the Arctic, including sea ice, pelagic water column, benthic habitats, beaches, surface waters, and macrobiota.^1−9^ Even though local sources can be regionally important, most plastic is thought to reach the Arctic by long-range transport with ocean currents.^2,10−12^ The West Spitsbergen current transports water masses from more temperate regions toward the Arctic and along the west coast of Svalbard. Monitoring of plastic on the deep-sea floor of this area indicated that plastic accumulated and reached densities comparable to areas west of Portugal (∼6600 items km^–2^).^3,13,14^

Several species in the Arctic, including many seabird species, were documented to ingest plastic.^6^ Among seabirds, northern fulmars (*Fulmarus glacialis*; hereafter referred to as fulmars) are particularly prone to ingest and accumulate marine plastic. This is partly explained by their feeding ecology as generalist surface-feeders in pelagic habitats and partly by the morphology of their stomach, consisting of two stomach compartments.^15^ The proventriculus (“forestomach”) is the first site where ingested food as well as marine litter is stored before it passes through a constriction into the ventriculus (hereafter referred to as “gizzard”).^16^ Because this constriction hinders regurgitation from the gizzard, indigestible hard items like plastic, once they reached the gizzard, can only be eliminated if they are small enough or after they are worn down to sizes small enough to pass to the intestine.^16^

Long-term monitoring of plastic pollution using large sample sizes of mainly beached fulmars from the North Sea showed that adult fulmars had consistently less plastic in their stomachs compared to younger individuals.^15,17^ This phenomenon is also known from other seabird species with similar anatomical attributes.^18−20^ A widely proposed explanatory hypothesis is that plastic is subjected to parental transfer, i.e., there is an offload of plastic from adults to their offspring along with regurgitated food from the proventriculus.^18,19,21^ The existence of this mechanism is well documented by plastic found in nestlings of several procellariiform seabirds that can only originate from parental feeding.^22−24^ Large quantities of ingested plastic in chicks and newly fledged birds were particularly found in flesh-footed shearwaters (*Ardenna carneipes*), short-tailed shearwaters (*Ardenna tenuirostris*), and Laysan albatrosses (*Phoebastria immutabilis*).^18,22,25−27^ Also, 2- and 6-week-old nestlings as well as fledglings of fulmars from the Faroe Islands were documented to contain plastic.^23,28−30^

Ryan^19,65^ hypothesized that parental transfer is the driving factor behind age-related differences in plastic burdens of a wide range of procellariiform seabirds.^19^ This hypothesis was among others based on an earlier study by Skira where progressively decreasing plastic loads in breeding adults of short-tailed shearwaters were documented throughout the breeding season.^31^ As part of his “annual cycle hypothesis,” Ryan further predicted that adult individuals have the lowest load of plastic in their stomachs after offloading to their chicks and then gradually reaccumulate plastic throughout the winter.^19^ Consequently, significant effects of plastic offload in adults would be temporarily limited to a specific season and restricted to those adults that successfully breed. Fulmar chicks are fed by both parents for 50–53 days before they fledge.^32^ After leaving their nests, fledglings weigh 115–119% of their parent’s weight making them practically flightless for a short period.^32^ Since these fledglings are not provisioned by their parents anymore, they start feeding themselves.^32^

In September, a mix of fledglings, immatures, and adults can be met at sea in proximity to fulmar breeding colonies. By simultaneously analyzing plastic burdens in fledglings and older fulmars (“nonfledglings” including older immatures and adults) directly after the chick-rearing period in Svalbard, we aimed to examine parental transfer in fulmars, by testing the prediction that fledglings have significantly more plastic in their stomachs than older fulmars.

## Materials and Methods

### Ethical Statement

To fully assess the stomach content of fulmars, it was necessary to sacrifice the birds because the anatomy of fulmar stomachs limits the possibility to obtain realistic proxies of plastic burdens from regurgitates or stomach flushing and other, nonlethal methods are not yet sufficiently developed.^33,34^ Due to logistical and financial limitations caused by the remoteness of the study site and the high density of scavengers, it was not possible to collect dead birds washed ashore like done in the North Sea region either.^35^ We targeted a sample size of 40 fulmars (final sample size obtained: 39 fulmars) based on a pilot study on marine litter monitoring with fulmars.^36^ The sampling was approved by the Governor of Svalbard (permit nr. 20/02252-2) and sampling methods were in accordance with the Norwegian animal welfare law and performed by skilled and licensed staff. We also maximized the scientific value of the collected birds by sampling as many tissues as possible for ecotoxicological research (not presented in this paper).

### Sampling Location and Protocol

Thirty-nine fulmars were collected at sea from a boat in Kongsfjorden (Svalbard; 78°55′N, 11°56′E), as part of a project registered in “Research in Svalbard” (RiS-ID 11562), between 8 and 11 September 2020. Flightless fledglings were caught using a D-shaped landing net with a telescopic rod and were sacrificed with a sharp blow to the head. A shotgun was used to collect older birds (nonfledglings). To prevent the loss of stomach content, we plugged the beaks with papers and used plastic cable ties to keep the beaks sealed. All birds were frozen at −20 °C within 1–4 h after the sampling in the fjord.

### Dissection

All fulmar dissections were performed in the laboratory following a standard protocol.^37,38^ During the dissections, the depth of the subcutaneous fat layer between the pectoral muscle and the skin was measured at its deepest with the depth rod of a vernier caliper. For this, the fat tissue was separated from the muscle tissue and kept attached to the skin on the side where it was measured. The gastrointestinal tracts (GITs) were dissected from the esophagus to the anus, along with several tissue samples for ecotoxicological research (not presented in this paper). New scalpel blades and gloves were used for each bird, and the tools were rinsed using soap, Milli-Q water, and ethanol.

### Aging and Sexing

Most fulmars in our sample set were fledglings that hatched approximately 50–60 days prior to sampling (53.8%). Birds of this age class were not able to fly during sampling and were confirmed as fledglings by the development state of their gonads (for males: small black testes; for females: small smooth ovaries without follicles), large bursa of Fabricius, and generally thick layers of subcutaneous fat.^37,38^

All females other than fledglings had gonads with follicles. While the oviducts of most females did not show any traces of former breeding, stretch markings in the surrounding tissues indicated former breeding activities in two females.^37^ Testes of older immature (i.e., individuals before the first breeding attempt) and adult males (individuals from breeding age on) cannot be distinguished by color, size, or shape outside the breeding season.^37^ All nonfledgling males in our sample set had bright, oval testes (average length × width = 29 mm ± 3 se). Because we lack sufficient information to distinguish between males before and after first breeding attempt, and to divide our sample set into two groups with similar sample sizes, we used the following age categories: “fledglings” and older fulmars or “nonfledglings” (which include all fulmars with fully developed gonads and may represent a mix of adults that did raise a chick in 2020, adults that skipped or failed breeding in 2020 and immatures). For the distribution of ages and sexes in our sample set, see Table 1.

**Table 1:** Overview over Sex and Age Distribution in Fulmars Sampled for This Study^a^

	total	females	males
total	39	22	17
fledglings	21	14	7
nonfledglings	18	8	10

a “Nonfledglings” include all fulmars older than fledglings.

### Plastic Extraction

The contents of all upper GITs and separately a subsample of 20 intestines (10 from fledglings and 10 from older birds) were transferred into glass beakers and a 10% solution of potassium hydroxide (KOH) was added to digest soft organic tissue.^39,40^ KOH was chosen for its efficiency to digest organic matter while preserving the mass, morphology, and the chemical integrity of many plastic polymers, even when heated up to 40 °C and shaken to 200 or 300 rpm, as evidenced by several studies.^41−43^ In this study, the beakers were kept on a low-profile shaker (IKA HS 501 digital, Staufen, Germany) at 100 rpm for at least 2 days (max 3 days) to enhance the digestion process, at room temperature. Thereafter, the mixtures of KOH solution and GIT content were filtered through a stainless-steel sieve (mesh size: 20 μm) and then vacuum-filtered through a filtering membrane (cellulose acetate filter, pore size 5 μm, Sartorius Stedim Biotech, Göttingen). The extracted particles were visually sorted, and only the plastic-like particles were further analyzed by spectroscopy. Particles from natural origin, e.g., squid beaks, exoskeleton of crustaceans, and other prey items that remained after KOH digestion as well as stones, etc., were not analyzed by FTIR spectroscopy, but thoroughly checked for hidden plastic particles. Filter papers were kept and can be used in future microplastic studies.

### Plastic Characterization: Length, Shape, Color, and Weight

The particles were placed on millimeter grid paper, assigned ID numbers, and photographed to enable detailed piece-by-piece characterization. The categorization of plastic by shape and color followed recent recommendations for standardizing the quantification of ingested plastic in marine megafauna.^44^ However, mass quantification was not performed for every characterization category. Numeric characterization data is included in the Supporting Information but does not account for possible fragmentation during lab handling (see the Discussion section).

All plastic items were either defined as industrial plastic (only pre-production pellets) or user plastics. Plastics of the latter category were further specified as fragments (decay products of bigger hard plastic pieces), sheets (remains of plastic bags and other soft plastic), threads (single fibers and bundles), or foams (mainly from polystyrene packaging).^44^ Particles that did not fit into any of these categories were grouped as “others” and reported along with further specifications. The color of each particle was determined visually by one observer (F.T.) from photographs and assigned one out of eight possible colors without the use of a color wheel. Greatest dimension was measured from photographs using the computer program ImageJ.^45^ To determine the total mass of plastic for each dissected bird, dry plastic items were weighed on an aluminum dish using a precision scale (Mettler Toledo ME104) with an accuracy of 0.0001 gram. When present, the mass of industrial pellets was determined separately.

### FTIR Analyses

Plastics were validated by determining polymer types using Fourier transform infrared (FTIR) spectroscopy (“Cary 630”) coupled to a Diamond Attenuated total reflectance (ATR) sampling accessory (Agilent Technologies, Santa Clara) similarly to previous studies.^23,46^ The analysis was performed with 32 scans and a resolution of 8 cm^–1^ at a wavenumber range of 650–4000 cm^–1^. Scans were collected after adjusting for background noise. A software program (microlab, Agilent Technologies) was used to automatically generate comparisons between the analyzed particles and standard spectra of reference materials, which were quantified with matching scores, an indicator for the similarity between the spectra. For this study, we used a threshold of 0.7 (1 would indicate a spectrum 100% identical to the reference spectrum) above which plastic polymers were accepted as identified. Prior to FTIR analyses, particles were bathed in ethanol and dried under a fume hood until all ethanol had evaporated. In cases where a polymer could not be identified after the first run (e.g., due to remaining biofilm or stomach oil), small parts of the material were sliced off to enable measurements from inner layers.

### Quantification Parameters

Plastic burden quantification is based on mass values as these are assumed to be of higher biological relevance than numbers of particles, which underly continuous fragmentation in the stomach and possibly during KOH digestion.^44^ Numeric data as well as particle sizes at the longest dimensions are primarily presented to discuss possible effects of our methodology. We report the following parameters: Average (mass, numbers) ± standard error (se), median (mass, numbers) with quartiles (*q*1, *q*3), and range, percentage of occurrence (PO), i.e., the percentage of birds for which plastic was found and the ecological quality performance (EcoQ %), i.e., the percentage of birds with a plastic mass ≥ 0.1 g.^37^ The EcoQ (%) was introduced by OSPAR (the convention for the protection of the marine environment of the North-East Atlantic) to monitor efforts to reduce marine plastic pollution toward the arbitrary set ecological quality objective that no more than 10% of fulmars should have a plastic mass ≥ 0.1 g in their stomachs.^15^

### Data Analysis

Statistical data analyses were performed with the statistics program R.^47^ First, a Shapiro–Wilk test was used to check whether the data were normally distributed. Since plastic mass values were not normally distributed (Shapiro–Wilk normality test: *w* = 0.658, *p* < 0.001), nonparametric tests were used to investigate if plastic burdens differed among age groups or sexes. Two-sided Wilcoxon rank sum tests were used for two sample comparisons. *P*-values of 0.05 were used as a significance threshold. For correlation analysis, we performed Spearman’s rank correlation tests in R (cor. test(*x*, *y*, method = “spearman”)).

## Results

### Plastic Burdens

Plastic was almost exclusively found in stomachs (including gizzard and proventriculus), while we only detected one single particle of plastic in the 20 intestines we examined. In total, we found 8.082 g of plastic in 39 individuals, with each bird having an average of 0.207 g ± 0.049 se (*q*1 = 0.026 g, median = 0.086 g, *q*3 = 0.250 g; Table 2; plastic burden details for each fulmar in the Supporting Information: Table S1). The total number of particles was 1408 (average ± se = 36.1 ± 10; *q*1 = 4; median = 21; *q*3 = 40), resulting in 0.0057 g per particle in average (see the Discussion section for possible fragmentation resulting from methodology). The particles had an average size of 5.5 mm ± 0.1 se at the greatest dimension (*q*1 = 3.4 mm; median = 4.6 mm; *q*3 = 6.2 mm), and only two pieces < 1 mm were detected.

**Table 2:** Overview over the Distribution of Plastic-Type Categories Found in Fulmar Samples^a^

	N	PO (%)	mass (%)	min (g)	*q*1 (g)	median (g)	*q*3 (g)	max (g)
total	1408	94.9	100	0	0.026	0.086	0.250	1.467
industrial plastics	38	48.7	8.8	0	0	0	0.030	0.138
user plastics	1370	94.9	91.2	0	0.026	0.077	0.213	1.414

a Industrial plastics are exclusively pre-production pellets (nurdles), while user plastics comprise several shapes that originate from plastic products. PO (%) is the percentage of occurrence.

Most particles were user plastics, dominated by fragments. Industrial pellets (nurdles) were found in less than half of the fulmars, with an average of 0.97 particle per bird (Table 2). An overview with numeric plastic characterization details, including shapes, polymer types, and colors, is included in the Supporting Material, but does not account for possible effects of KOH digestion (Table S2).

The EcoQO threshold of 0.1 g plastic was exceeded in 46.2% of the individuals. Only two fulmar stomachs (both from adults) did not contain plastic. Our data revealed that plastic burdens differed significantly between the age classes (*w* = 327, *p* < 0.001) with the highest loads in fledglings and lowest loads in nonfledglings (Table 3).

**Table 3:** Overview over Plastic Burdens (Mass in Gram) among Different Age Categories of Fulmars^a^

	N	PO (%)	EcoQ (%)	average (g)	se	min (g)	*q*1 (g)	median (g)	*q*3 (g)	max (g)
total	39	94.9	46.2	0.207	0.05	0	0.026	0.086	0.25	1.467
fledglings	21	100	66.7	0.34	0.08	0.016	0.085	0.171	0.404	1.467
nonfledglings	18	88.9	22.2	0.053	0.015	0	0.005	0.026	0.077	0.211

a The sample size (*N*) within each category is reported. Averages are given with standard error (se) and medians with ranges (min, max) and quartiles (*q*1, *q*3). PO = percentage of occurrence, EcoQ (%) = percentage of birds with >0.1 g plastic.

**Figure 1:**
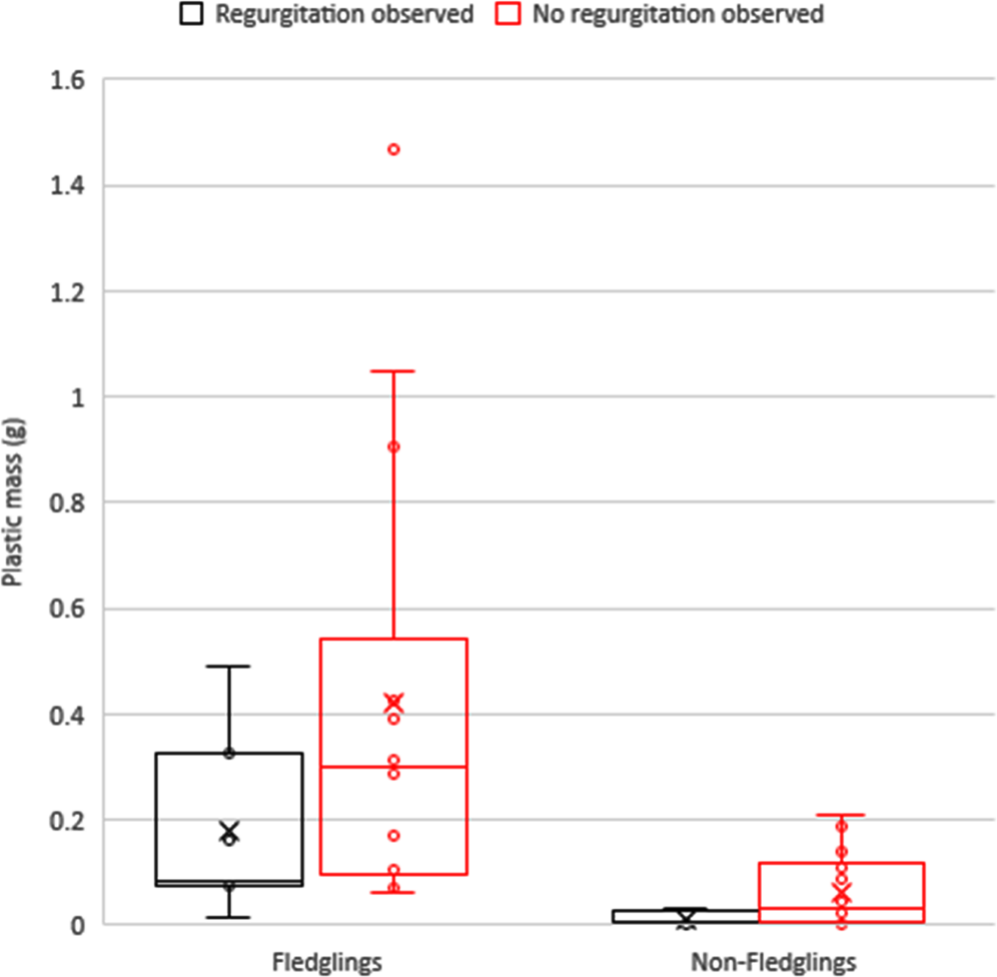
Comparison between plastic mass of fulmars that were observed regurgitating and those that were not observed regurgitating during sampling within the two age categories “fledglings” and “nonfledglings”.

During sampling, 28% of fulmars were observed regurgitating stomach content. Among all fulmars, fledglings were observed to regurgitate the most (33%) while other fulmars were less frequently observed to do so (22%). Plastic mass and consequently EcoQs were lower in regurgitating fulmars compared to the birds which did not regurgitate (27.3 vs 53.6%). This pattern was seen for the whole sample and within the different age categories (fledglings: 42.9 vs 78.6%, in nonfledglings 0 vs 28.6%; Figure 1). However, none of these differences were statistically significant (total sample set: *w* = 192.5, *p* = 0.236; fledglings: *w* = 67, *p* = 0.197; older fulmars: *w* = 42.5, *p* = 0.137).

Similarly, plastic mass tended to be higher in females compared to males. However, the significance threshold was just missed (*w* = 255, *p* = 0.058) despite females being overrepresented in higher burdened fledglings and underrepresented in the little burdened nonfledglings (Supporting Information: Figure S1).

### Subcutaneous Fat Layer Depth

**Figure 2:**
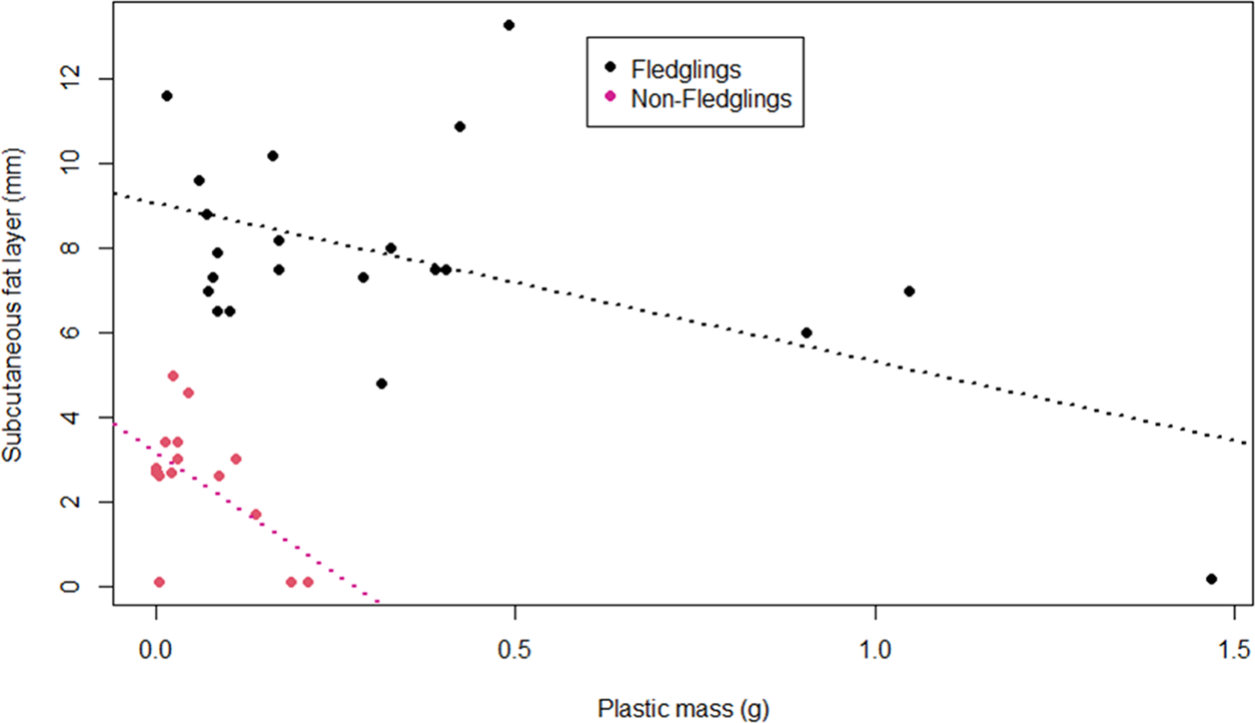
Visualization of the relation between mass of ingested plastic (g; *x*-axis) and depth of the subcutaneous fat layer (mm; *y*-axis). Each dot indicates an individual and the age category is indicated by colors. Linear regressions (dotted lines) are included for fledglings (black) and nonfledglings (violet-red).

The depth of the subcutaneous fat (SF) layer was examined in relation to the mass of ingested plastic (Figure 2). SF differed significantly between fledglings and nonfledglings (*w* = 322, *p* < 0.001). Therefore, analyses were run separately for each age category (Figure 2). We found nonsignificant negative correlations between plastic mass and SF layer depth in fledglings (Spearman’s rank correlation: rho = −0.273, *p* = 0.232) and in older birds (rho = −0.217, *p* = 0.420).

## Discussion

### Methodology

Our methodology deviated from the standard protocol for plastic studies in fulmars using a sieve with a smaller mesh size (20 μm instead of 1 mm) and by digestion with a 10% solution of KOH on a shaker.^37^ While KOH digestion of plastic ingested by marine biota indicated that the integrity of plastic particles is largely maintained in many polymers, further fragmentation of plastic particles by this methodology cannot be excluded.^39,41−43^ The mass/number ratio of plastic particles can be an indicator of the degree of fragmentation. This average weight per particle was higher in other studies; e.g., in data from the North Sea (1980: 23.3 mg; 1995–2007: 10.2–19.6 mg; 2014–2018: 12.1 mg) compared to our study (5.7 mg).^15,17^ On the other hand, our data align with a former study from Svalbard, where standard protocol was followed (average mass/*n* = 5.2 mg), indicating that highly degraded marine plastic fragments in fulmar stomachs are characteristic for Svalbard.^35^ This is further supported by comparing our study with a study on fulmar chicks from the Faroe Islands, where the same KOH digestion method was used and the average and median weight per particle were higher compared to our data from Svalbard (mass/number ratio average = 12.1 mg; median = 10.7 mg).^23^

Despite the small mesh size we used, we detected only two particles < 1 mm at the longest dimension (both > 0.8 mm). Therefore, we assumed that the impact of our methodology on mass quantification parameters was negligible. Also, the percentage of fulmars where plastic was found (PO = 94.9%) would not have changed, when excluding these two particles from our study.

### Plastic Burdens Contrasted between Fledglings and Older Fulmars

Our study documented significant differences between plastic burdens of the two age categories, with more plastic in fledglings compared to older fulmars. Since the fulmars were caught directly after the chick-rearing season, this difference supported that high quantities of plastic were transferred from parent fulmars to their chicks. Among the 18 older fulmars, two were found without plastic and four with plastic burdens < 0.01 g. However, there was a high variability of plastic burdens within this age category, which likely consisted of individuals with and without breeding activity in 2020, ergo with and without offloading of plastic to their chicks. It is assumed that less than half of adult fulmars successfully raise a chick in each breeding season, and our category “nonfledglings” did likely represent a mix of adults and older immatures.^32,48^

Effects of parental transfer are important to consider when using fulmars as bioindicators for marine plastic pollution, especially in short-term incidental studies with small sample sizes in a time frame close to breeding season and in proximity to colonies. The extent to which these studies can be constrained by these effects would vary with sampling season, age composition, and further depend on two unknown aspects: The retention time of parentally delivered plastics in juvenile fulmars and the time it takes for adults to reaccumulate plastics after offloading to chicks.

The factors season and age can also impact plastic burdens of fulmars beyond parental transfer. Seasonal variation of plastic loads in procellariiform seabirds was hypothesized to result from migration between higher-polluted winterfeeding grounds and less-polluted breeding areas, where the birds subsequently eliminated their imported plastics.^17,49−51^ Such a decrease of plastic throughout the breeding season was also observed in fulmars in the Canadian Arctic but may have been caused by a higher proportion of higher burdened nonbreeders residing at the colonies earlier in breeding season.^17,51^

Estimates for the retention time of plastics in fulmars or other procellariiform seabirds are under debate and range from 1 month to several years, and it is likely that retention times also depend on plastic types.^50,52−56^ The grinding efficiency could also be less developed in early life stages of fulmars.^57^ In the Faroe Islands, plastic loads in fulmars were documented to remain on a high level throughout their first and second years, with a gradual decrease for each higher age class.^30^ However, the high burdens observed in young age classes, several months after fledging, unlikely originate from parental transfer based on findings from beached juvenile fulmars at the Pacific coast of the United States (Oregon and Washington).^48^ Among 156 juvenile fulmars (3–6 months after fledging) found between 2008 and 2015, 20 juveniles did not contain plastic and high proportions of plastic mass in the proventriculi of the other juveniles indicated large quantities of recently ingested plastic.^48^ High plastic loads in juveniles were therefore suggested to be linked to higher ingestion rates of naïve foragers, mistaking plastics for prey species.^48,58^

### Role of Regurgitation

Alternatively, van Franeker et al. suggested that the gradual decrease of plastic burdens with higher age classes can result from that older fulmars spend more time at land in the colonies.^59^ Older immatures already start to establish nest-sites years before their first breeding attempt, where they may eject plastic along with defensive spitting of stomach oil.^59^ Stomach oil is primarily used as an energy reserve but also as a weapon against nest competitors, intruders, and predators.^32,59^

In our study, we found that the average and median of plastic mass tended to be lower in fulmars that regurgitated prior to or during sampling compared to fulmars that did not regurgitate. Although not statistically significant, this aspect should not be overlooked as this pattern was consistent in both age groups (i.e., fledglings and nonfledglings). Furthermore, sample sizes were small and regurgitation effects could only be examined using a simple presence/absence approach. Interestingly, the fledgling that contained the least plastic (0.016 g) was observed regurgitating extensively three times.

Among procellariiform seabirds, chicks of flesh-footed shearwaters (*A. carneipes*) and Laysan albatrosses (*P. immutabilis*) were documented to regurgitate considerable amounts of plastic along with other indigestible items prior to leaving their nests.^25,27^ Such a behavior is not documented in fulmar chicks; however, they can regurgitate spontaneously at their nests.^60^ Such regurgitates from fulmar nestlings (*N* = 14) were opportunistically collected in Ireland and analyzed for plastic.^60^ The regurgitates contained an average plastic mass of 0.013 g (range: 0–0.1043 g, se ± 0.032) and an average number of 0.5 piece (range: 0–3, sd ± 0.90) while plastic was found in 28.7% of these samples.^60^ In a rehabilitation center, three fulmars (of unknown age) were documented to regurgitate plastic in high quantities (6.669–10.591 g or 22–74 pieces).^56^

### Implications for Fulmars as Bioindicators for Marine Plastic Pollution

As a result of the age composition of fulmars and the sampling season, we cannot conclude on regional plastic pollution levels or temporal changes by comparisons with other studies. When only considering birds older than fledglings, the EcoQO performance would be 22.2%, similar to a previous study from Svalbard (EcoQ = 22.5%) that did not include juveniles.^35^ On the other hand, the former study from Svalbard was performed a few weeks later after chick-rearing, where adults may have reaccumulated plastics, following the annual cycle hypothesis.^19,35^

To detect regional differences and temporal changes in marine plastic pollution using fulmars as indicators, it is desirable to use homogeneous age groups from the same season.^19^ Because fulmar fledglings are traditionally harvested for consumption in the Faroe Islands, Iceland, and to a lesser degree in Greenland, the potential of cooperating with hunters should be further explored.^28−30,61,62^ This approach would allow us to assess the relative exposure of fulmars to plastic across some of the remote regions of the North Atlantic and avoid age and season as confounders (ideally without sacrificing birds for science). So far, there is published data for fulmar fledglings hunted by locals in the Faroe Islands in the years 2005–2009.^30^ Interestingly, the average plastic mass in these fledglings was on a similar level compared to our findings, despite Svalbard being at a 78° northern latitude against 62° for the Faroe Islands.^30^ Even lower plastic burdens in Faroese fledglings were reported in studies that did not primarily aim to quantify but to characterize or analyze ecotoxicological aspects of plastics.^28,29^ This contrasts with an otherwise documented decrease in plastic mass in fulmar stomachs with higher latitudes.^15,17^ On the other hand, the data from Faroese fledglings may have been an underestimate resulting from handling by the hunters.^29^ In the light of the underexplored role of regurgitation, it is also possible that plastic loads in fulmar fledglings at different locations are determined by different frequencies of defensive stomach oil spitting trigger events resulting from different densities of predators or disturbance by human.

### Health Impairments

Plastic particles were witnessed perforating the GIT walls in two cases. The first case was observed in a fledgling that had a sharp plastic fragment horizontally stuck in the proventriculus, which likely created a hole in the proventricular lining. This fledgling was observed regurgitating during sampling without ejecting this large fragment (∼20 mm) from the proventriculus. A similar sharp fragment was also reported being possibly linked to a hole in the proventriculus of a flesh-footed shearwater.^25^ The second case was a single string thread that was witnessed (by F.T. and F.C) perforating the intestine. During the dissection, the bright green tip of this thread was seen outside the intestine, while the remaining part was still inside. This thread was also the only piece of plastic we found outside the two stomach compartments. However, the GIT of this fulmar might have been damaged by a shot during sampling. Although it was perceived differently during the dissection, it cannot be excluded that the intestine lining was punctured by a shot. In general, damages of the GIT walls linked to plastic pieces are rarely reported for fulmars, considering the high numbers that are dissected for stomach analyses each year. A recent pathology report on a large sample size of fulmars (173) beached on Sable Island (Canada) did not suggest direct adverse health effects linked to plastic ingestion, but starvation as the most common cause of death.^63^

Negative correlations between subcutaneous fat layer depth and ingested plastic mass in fledglings and nonfledglings were insignificant. In fledglings, it was however notable that the individual with the highest plastic burden by mass (1.467 g) had barely any subcutaneous fat reserves left, while all other fledglings had a remarkable fat layer (in mm: min = 4.8; *q*1 = 7; median = 7.5; *q*3 = 9; max = 13.3). During the dissection of the most burdened fledgling, we found a ball bearing (“BB”) bullet (acrylonitrile butadiene styrene; 5.6 mm) blocking the transition from the gizzard to the intestine. This bullet may have thereby reduced nutrient intake and hindered fat deposition.

Fat deposition can be negatively affected by reduced stomach capacity associated with reduced efficiency of nutrient intake.^26,57^ Theoretically, high volumes of plastic can also prevent the contraction of the gizzard, which otherwise would trigger appetite, possibly reducing foraging efforts.^57,64^ However, an estimate for the maximum capacity of plastic in the gizzard is suggested to be ∼2.6 g, i.e., almost twice the plastic load of the highest burdened fulmar in our study (1.467 g).^48^

A feeding experiment on chicken (*Gallus gallusDomesticus*) provided evidence for the negative impacts of plastic on growth and development.^65^ Similar impacts on development and body mass were also found in a study on fledglings of flesh-footed shearwaters from Lord Howe Island (Australia).^26^ Here, the negative correlation between body mass and plastic ingestion was suggested to result from a reduced stomach capacity and subsequently reduced nutrient uptake.^26^ Four of these fledglings (*N* = 34) with an average plastic mass of 21 g ± 8 fell below a body mass threshold established for survival throughout the first year at sea for similarly sized sooty shearwaters (*Ardenna grisea*).^26^ Even though plastic masses were much higher in that study, compared to the plastic masses we found in fulmar fledglings (even when considering size differences between those two species), it indicates that plastic ingestion in high quantities can have the potential to increase juvenile mortality in a species.

While direct physical injuries caused by plastic ingestion are relatively uncommon and cause–effect relationships between plastic ingestion and body conditions are hard to prove in the field, plastic-related contaminants have obtained increasing attention by the scientific community. Some plastic additives are known to leach out from the plastic material once in the environment or once ingested.^29,66−72^ Finally, research on the occurrence of microplastics in other tissue than the stomach content of seabirds is scarce. A recent study evidenced that microplastics could have more severe impacts than previously thought, highlighting the need for complementary research toward associated contaminants and histological impacts of plastic ingestion and translocation.^73^

## Supporting Information Available

Bird characteristics and plastic burdens per bird (Table S1); details for plastic characteristics (shapes, polymers, colors) (Table S2); and comparison of plastic mass between sexes within age groups (Figure S1) (PDF)
