# Supplementary material for: Implications of Regurgitative Feeding on Plastic Loads in Northern Fulmars (Fulmarus glacialis): A Study from Svalbard
Source: Environ Sci Technol. 2023 Feb 22;57(9):3562–70. doi: 10.1021/acs.est.2c05617 (PMC9996815; doi:10.1021/acs.est.2c05617)
Supplement: Supplementary file 1 — es2c05617_si_001.pdf [file es2c05617_si_001.pdf]

Implications of regurgitative feeding on plastic loads in Northern Fulmars (*Fulmarus glacialis*): A study from Svalbard

Felix Tulatz[1,2*], Geir Wing Gabrielsen[2], Sophie Bourgeon[1], Dorte Herzke[1,3], Rupert Krapp[2], Magdalene Langset[4], Svenja Neumann[2], Anna Lippold[2], France Collard[2]

1: UiT - The Arctic University of Norway, Department of Arctic and Marine Biology, N-9037 Tromsø, Norway; 2: Norwegian Polar Institute, Fram Centre, N-9296 Tromsø, Norway; 3: Norwegian Institute for Air Research, Fram Centre for Climate and the Environment, Fram Centre, N-9296, Tromsø, Norway; 4: Norwegian Institute for Nature Research, Høgskoleringen 97034 Trondheim, Norway

***Corresponding author: felix-tulatz@hotmail.com**

Summary
Pages:5; Tables: 2; Figures: 1

Table of contents

Table S1. Bird characteristics and plastic burdens per bird.

Table S2. Details for plastic characteristics (shapes, polymers, colors).

Figure S1. Comparison of plastic mass between sexes within age groups.

Table S1. Information about bird characteristics and plastic burdens per bird for fulmars sampled in Kongsfjorden (Svalbard) in September 2020. The age categories are *Fledglings* (50-60 days old), and *Non-Fledglings* (all fulmars older than fledglings).

Fulmar ID	Age	Sex	Subcutaneous fat depth (mm)	Plastic mass (g)	User plastic mass (g)	Industrial plastic mass (g)
SVA-2020-001	Fledgling	F	6	0.9043	0.8567	0.0476
SVA-2020-002	Fledgling	F	7.5	0.1707	0.1459	0.0248
SVA-2020-003	Fledgling	F	4.8	0.3135	0.2599	0.0536
SVA-2020-004	Fledgling	F	13.3	0.4905	0.4728	0.0177
SVA-2020-005	Fledgling	F	7.5	0.3889	0.3219	0.067
SVA-2020-006	Fledgling	F	10.9	0.4235	0.3849	0.0386
SVA-2020-007	Fledgling	F	7.5	0.4043	0.381	0.0233
SVA-2020-008	Fledgling	F	7.3	0.0801	0.0801	0
SVA-2020-009	Fledgling	F	6.5	0.0851	0.0661	0.019
SVA-2020-010	Non-Fledgling	M	2.6	0.0881	0.077	0.0111
SVA-2020-011	Fledgling	F	10.2	0.1623	0.127	0.0353
SVA-2020-012	Non-Fledgling	M	2.8	0	0	0
SVA-2020-013	Fledgling	F	7	1.0477	0.9935	0.0542
SVA-2020-014	Non-Fledgling	F	0.1	0.2113	0.0729	0.1384
SVA-2020-015	Fledgling	M	0.19	1.4667	1.414	0.0527
SVA-2020-016	Non-Fledgling	M	3	0.0305	0.0305	0
SVA-2020-017	Fledgling	F	7.9	0.086	0.0778	0.0082
SVA-2020-018	Fledgling	M	8	0.326	0.2864	0.0396
SVA-2020-019	Non-Fledgling	F	2.7	0	0	0
SVA-2020-020	Fledgling	M	8.8	0.0702	0.0505	0.0197

SVA-2020-021	Fledgling	M	7	0.0741	0.0695	0.0046
SVA-2020-022	Non-Fledgling	M	NA	0.0018	0.0018	0
SVA-2020-023	Non-Fledgling	M	5	0.023	0.023	0
SVA-2020-024	Non-Fledgling	F	0.1	0.1872	0.1598	0.0274
SVA-2020-025	Non-Fledgling	M	3	0.1117	0.1117	0
SVA-2020-026	Non-Fledgling	F	2.7	0.0025	0.0025	0
SVA-2020-027	Fledgling	M	8.2	0.1703	0.1703	0
SVA-2020-028	Non-Fledgling	F	3.4	0.0296	0.0296	0
SVA-2020-029	Non-Fledgling	F	NA	0.0315	0.0315	0
SVA-2020-030	Non-Fledgling	M	3.4	0.0142	0.0142	0
SVA-2020-031	Non-Fledgling	M	2.7	0.0223	0.0223	0
SVA-2020-032	Non-Fledgling	F	1.7	0.1399	0.1399	0
SVA-2020-033	Non-Fledgling	M	4.6	0.0455	0.0455	0
SVA-2020-034	Non-Fledgling	F	0.1	0.0053	0.0053	0
SVA-2020-035	Fledgling	M	6.5	0.1037	0.1037	0
SVA-2020-036	Fledgling	M	9.6	0.0607	0.0607	0
SVA-2020-037	Fledgling	F	11.6	0.0156	0.0156	0
SVA-2020-038	Non-Fledgling	M	2.6	0.0045	0.0045	0
SVA-2020-039	Fledgling	F	7.3	0.2888	0.2566	0.0322

Table S2. Characterization details for plastic particles (> 1mm at longest dimension) found in fulmars sampled in Svalbard 2020. Note that numeric data (n) may have been impacted by methodology (see discussion). Percentage of Occurrence (PO%) is the % of fulmars where a characterization category was found. Average, median, quartiles and maximum are per

fulmar, minimum for all categories is zero. The four plastic pieces in the shape category "others" were two cylindric hollow plastic beads and two "BB bullets". The plastic polymers included: PE = polyethylene, PP = polypropylene, PS = polystyrene, PET = polyethylene terephthalate, ABS = acrylonitrile butadiene styrene, PU = polyurethane and PA = polyamide.

	PO %	N	Relative abundance %	Average ± SE	Q1	Median	Q3	Maximum
Category								
Pellets	48.7	38	2.7	1 ± 0.2	0	0	1.5	5
Fragments	89.7	1126	80.1	28.8 ± 8.8	4	17.5	30.5	333
Threads	56.4	100	7.1	2.6 ± 0.7	0	1	3	18
Sheets	69.2	95	6.8	2.4 ± 0.7	0	1	2	25
Foams	28.2	43	3.1	1.1 ± 0.4	0	0	0	14
Others	7.7	4	0.3	0.1 ± <0.1	0	0	0	2
Polymer type								
PE	87.2	983	70	25.2 ± 7.6	2	15	26.5	291
PP	79.5	374	26.6	9.6 ± 2.4	1.5	6	11	90
PS	30.8	44	3.2	1.1 ± 0.5	0	0	1	14
PET	2.6	1	< 0.1	< 0.1	0	0	0	1
ABS	2.6	1	< 0.1	< 0.1	0	0	0	1
PU	2.6	1	< 01	< 0.1	0	0	0	1
PA	5.1	2	< 0.1	< 0.1	0	0	0	1

Color								
White	74.4	169	12	4.3 ± 1.2	0.5	2	4.5	35
Grey	33.3	37	2.6	0.9 ± 0.3	0	0	1	9
Black	53.8	54	3.8	1.4 ± 0.3	0	1	2	6
Blue-Purple	43.6	29	2.1	0. ± 0.2	0	0	1	6
Green	41	33	2.3	0.8±0.3	0	0	1	8
Orange-Brown	59	119	8.5	3.1 ± 1	0	1	3.5	36
Red-Pink	41	34	2.4	0. 9 ± 0.3	0	0	1	9
Yellow	84.6	931	66.2	23.9 ± 7.3	2	13	25	278

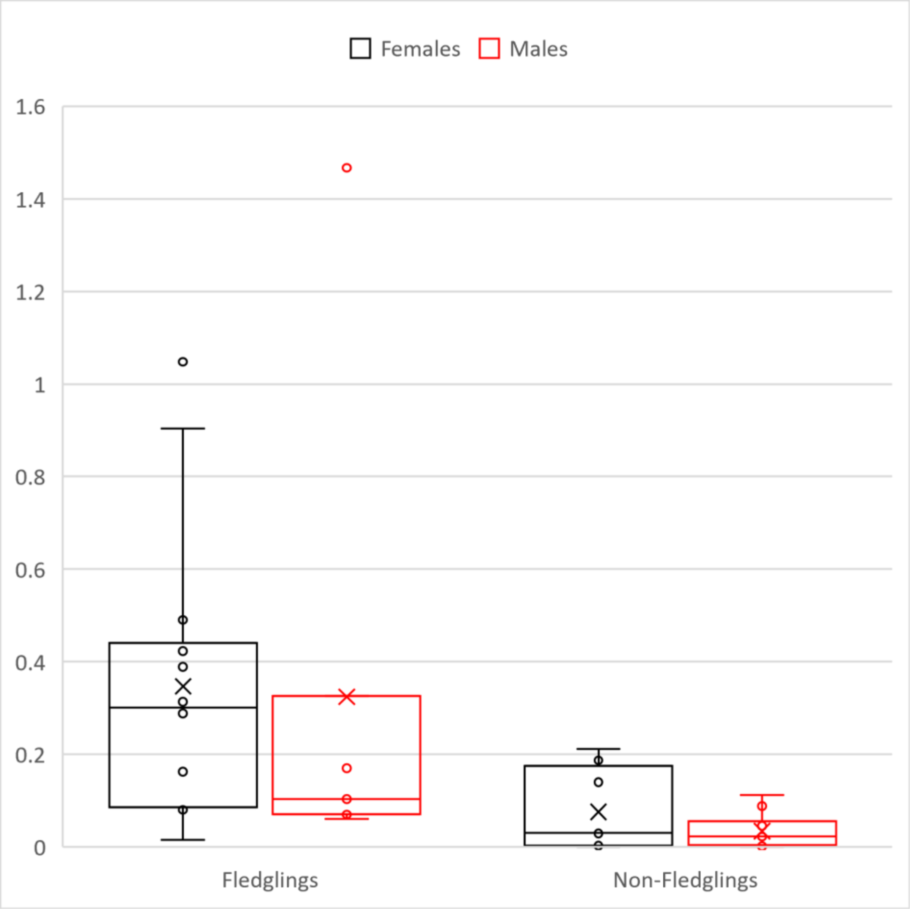

Figure S1. Boxplots comparing mass of plastic between sexes within the age categories *Fledglings* and *Non-Fledglings*. The horizontal lines inside the boxes mark the median, and the lower and upper boundary lines of the boxes indicate the first and the third quartiles, respectively. Dots beyond the whiskers represent outliers.
